# Female-specific decreases in alcohol binge-like drinking resulting from GABA_A_ receptor delta-subunit knockdown in the VTA

**DOI:** 10.1038/s41598-019-44286-0

**Published:** 2019-05-30

**Authors:** L. M. Darnieder, L. C. Melón, T. Do, N. L. Walton, K. A. Miczek, J. L. Maguire

**Affiliations:** 1 Graduate Program in Neuroscience, Sackler School of Graduate Biomedical Sciences, Tufts University, Boston, MA 02111 USA; 2 Tufts University School of Medicine, Department of Neuroscience, Boston, MA 02111 USA; 3 Tufts University, Psychology Department, Medford, MA 02155 USA; 4 Northeastern University, Bouvé College of Health Sciences, Boston, MA 02115 USA; 5 University of Massachusetts Boston, Honors College of Nursing and Health Sciences, Boston, MA 02125 USA

**Keywords:** Ion channels in the nervous system, Neurotransmitters

## Abstract

Binge drinking is short-term drinking that achieves blood alcohol levels of 0.08 g/dl or above. It exhibits well-established sex differences in GABAergic inhibitory neurotransmission, including extrasynaptic δ subunit-containing GABA_A_ receptors (δ-GABA_A_Rs) that mediate tonic inhibition, or synaptic γ2-containing GABA_A_Rs which underlie fast, synaptic, phasic inhibition have been implicated in sex differences in binge drinking. Ovarian hormones regulate δ-GABA_A_Rs, further implicating these receptors in potential sex differences. Here, we explored the contribution of extrasynaptic δ-GABA_A_Rs to male and female binge-like drinking in a critical area of mesolimbic circuitry—the ventral tegmental area (VTA). Quantitative PCR revealed higher *Gabrd* transcript levels and larger tonic currents in the VTA of females compared to males. In contrast, male and female *Gabrg*2 transcript levels and measures of phasic inhibition were equivalent. Intra-VTA infusion of AAV-Cre-GFP in floxed *Gabrd* mice downregulated δ-GABA_A_Rs and decreased binge-like drinking in females. There was no significant difference in either male or female mice after GABA_A_R γ2 subunit reduction in the VTA following AAV-Cre-GFP infusion in floxed *Gabrg2* mice. Collectively, these findings suggest sex differences and GABA_A_R subunit specificity in alcohol intake.

## Introduction

Binge drinking, or drinking intoxicating doses of alcohol yielding blood alcohol levels ≥0.08 g/dL^1^, may precipitate alcohol use disorders. Men have higher rates of binge drinking^2^, but rates are increasing in women^3^. Though potential sex differences in the trajectory of alcohol use to alcohol-use related problems have been reported^4^, it is unclear whether or how sex may impact the mechanisms underlying binge drinking behavior.

Rodent models of binge-like drinking support sex differences in this pattern of alcohol intake^5–7^ and offer an opportunity to clarify whether sex-specific mechanisms drive this pattern of alcohol intake. The GABAergic system may be particularly important since alcohol potentiates the inhibitory effects of GABA^8^ and a subclass of GABA_A_ receptor subunits have been proposed to confer sensitivity to the low-to-moderate doses of alcohol characteristic of the start of an active binge drinking session^9,10^. GABA receptors containing the γ2 subunit, which mediate phasic inhibition, respond to high alcohol concentrations; whereas, δ-containing GABA_A_ receptors, which mediate tonic inhibition, have been suggested to respond to low/moderate alcohol concentrations^11–13^. The role of extrasynaptic GABA_A_Rs in conferring sensitivity to alcohol has been proposed to be due to direct effects on these receptors^14^, but may also be mediated indirectly via changes in presynaptic GABA release^15–17^ or ethanol-induced increases in neurosteroids^18–20^ that potentiate the effects of GABA on these extrasynaptic receptor subtypes^21^. Whether the δ subunit is necessary for the behavioral effects of alcohol concentrations achieved in an active binge drinking session remains unknown, although the global loss of these receptors has been shown to reduce alcohol intake and alcohol preference^12^.

Interestingly, δ-containing GABA_A_Rs have also been shown to be regulated by ovarian hormones^22–24^, potentially implicating these receptors in sex differences in alcohol use. In fact, *Gabrd* transcript levels are altered in the ventral tegmental area (VTA) across the estrous cycle^24^; however, the role of these receptors in potential sex differences remains unexplored and is, thus, the focus of the current study. GABAergic signaling in the VTA regulates mesolimbic reward circuitry by modulating activity of dopaminergic (DAergic) projections from the VTA to the limbic forebrain, particularly the nucleus accumbens and, as such, is critical to alcohol intake and preference. Activity of this VTA DAergic projection is also estrous and sex-dependent^25^ and may be differentially sensitive to the GABAergic effects of alcohol. Therefore, we hypothesized that the expression of GABA_A_R δ subunits in this area may play a role in sex differences in binge-like alcohol intake. Consistent with this hypothesis, microinjection of a preferential agonist at δ-containing GABA_A_Rs (4,5,6,7-tetrahydroisoxazolo(5,4-c)pyridin-3-ol; THIP) into the posterior VTA (pVTA) reduced binge-like drinking in female mice^24^.

Here, we demonstrate sex differences in *Gabrd* mRNA and δ-mediated tonic inhibition in the VTA, with higher levels in observed in female mice compared to males. Knockdown of the *Gabrd* gene in the pVTA decreased binge-like drinking for females but not males; whereas, knockdown of *Gabrg2* gene had no effect in either sex. Collectively, this work provides evidence for a unique role for the δ subunit in low-dose alcohol consumption, underscoring its importance in the VTA of female mice and implicating these receptors in sex differences in binge-like drinking.

## Materials and Methods

### Animals

Floxed *Gabrd* mice were generated and previously characterized by our laboratory (Lee & Maguire 2013). Floxed *Gabrg2* mice were acquired from The Jackson Laboratory (Stock #016830). All mice used in the current study were adults (8–14 weeks of age) and were maintained in-house at the Tufts University School of Medicine, Division of Laboratory Animal Medicine and were handled according to protocols approved by the Tufts University Institutional Animal Care and Use Committee. Given the already published findings demonstrating estrous cycle changes in GABA_A_R δ subunit expression in the VTA^24^, all female mice used in the current study were acyclic as previously established by our group and others^26,27^.

### Stereotaxic surgery

Adult male and female floxed *Gabrd* and *Gabrg2* mice were stereotaxically injected with either AAV2-GFP or AAV2-Cre-GFP (Vector Biolabs) into the VTA using the following coordinates: A/P −3.6 mm, M/L ±0.5 mm, D/V 4.5 mm. This approach results in expression of GFP or Cre recombinase in a subset of neurons in the VTA.

### Drinking in the dark

Approximately two weeks after surgery, mice were individually housed on a reverse light/dark cycle (12:12 h, lights off at 0700). One week later, they were subjected to the four-day Drinking in the Dark (DID), binge-like drinking protocol established by Rhodes *et al*.^28^. Volume differences across each access period were converted to individual g/kg values for each mouse based on mouse weights obtained on the day prior to DID start.

### BEC measurements

Blood ethanol concentrations (BECs) were measured on a subset of *Gabrg2* and *Gabrd* DID mice. Submandibular blood samples were obtained immediately after the end of the 4 h binge session and plasma was isolated. An AM1 Alcohol Analyzer (Analox Instruments, Lunenburg, MA) was used to obtain all BEC values. Samples were run in duplicate and averaged to obtain a final value.

### Sucrose preference and quinine aversion

A subset of mice were also subjected to successive sucrose preference and quinine aversion testing. Briefly, mice were provided access to either 2% (w/v) sucrose or normal drinking water. Bottle weights were obtained every 24 h (1200 each day) for four, consecutive days. The position of the bottles was alternated after each 24 h period to avoid side preference. The volume (mL) of water consumed was also calculated (1g_H2O_ = 1mL_H2O_) and consumption differences were used to calculate preference scores according to the following formula: (g_sucrose_/[g_sucrose_ + g_water_]). After three days of water access, mice were subjected to quinine aversion testing using the same procedure, but with 0.3 mM quinine monohydrochloride solution and water.

### Immunohistochemistry and image analysis

GFP immunofluorescence was performed to enhance the GFP expression following stereotaxic injection of AAV-GFP and AAV-Cre-GFP. VTA slices (40 µm) were blocked with 10% normal goat serum (NGS) and incubated with a polyclonal anti-GFP primary antibody (rabbit, 1:1000, Invitrogen, Waltham, MA, USA) overnight at 4 °C and incubated with a polyclonal AlexaFluor 488 secondary antibody (rabbit, 1:200, Invitrogen, Waltham, MA, USA) for 2 h at room temperature. GFP immunostaining was imaged on a Keyence BZ-X700 (Keyence, Itasca, IL, USA) and analyzed using the Fiji distribution of ImageJ (NIH). Images were converted to binary masks prior to analysis and Integrated Density values were used to define signal intensity within a region of interest. Binary masks cause inversion of pixel values, thus greater ID indicates lower GFP signal.

### Real-time quantitative PCR (qPCR)

Sex-specific, baseline *Gabrd* and *Gabrg2* transcript levels were compared between ethanol naïve wildtype males and females. Similar experiments were conducted on floxed *Gabrd* and *Gabrg2* mice bilaterally infused with either AAV-GFP or AAV-Cre-GFP to assess gene knockdown.

RNA was isolated from the VTA and hippocampus using a QIAGEN RNeasy Mini Kit according to the manufacturer’s instructions (Frederick, MD, USA) and the concentration and integrity was analyzed using an Agilent Genomics 2100 Bioanalyzer (Santa Clara, CA, USA). All samples with RNA integrity numbers (RIN) above 8.0 indicated highly intact RNA and were used to generate cDNA. Reverse transcription was performed using SuperScript III First-Strand Synthesis System according to the manufacturer’s instructions (ThermoFisher Scientific, Cambridge, MA, USA). SYBR-green-based qPCR was performed using a Stratagene Mx3000P (Agilent Genomics, Santa Clara, CA, USA) with 3 ng of each VTA cDNA template and primers listed below (Table 1) using the following thermocycling protocol: 10 min ramp-up cycle to 95 °C, 40 cycles of 30 sec at 95 °C, 1 min at 55 °C, and 30 s at 72 °C, and a final dissociation curve at 95 °C for 1 min, 55 °C for 30 sec, and 95 °C for 30 sec. All reactions were performed in triplicate and a final, averaged C_q_ value used for analysis. A C_q_ value was excluded only if it was greater or less than 1 cycle relative to the remaining C_q_ values. Relative transcript levels were normalized first to the reference gene β-actin and the relative changes in expression levels compared to controls were calculated according to the 2^−ΔΔct^ method (Livak & Schmittgen 2001). For the comparison between males and females, the transcript levels were normalized to the females since the Gabrd transcript levels were significantly higher in this group. For the knockdown experiments, each group was normalized to the AAV-GFP controls.

**Table 1:** List of primers used for qRT-PCR. The primers used to detect baseline Gabrd and Gabrg2 transcript levels are designated as WT. The primers used to detect the Cre-mediated excision are designated “Cre excision”. Gabrd and Gabrg2 transcript levels were normalized to β-actin.

Gene	Forward Primer (5′ to 3′)	Reverse Primer (5′ to 3′)
*β-actin*	GGC TGT ATT CCC CTC CAT CG	CCA GTT GGT AAC AAT GCC ATG T
*Gabrd* (*WT*)	ATT GGG GAC TAC GTG GGC T	CCA CAT TCA CAG GAG CAC C
*Gabrd* (*Cre excision*)	GGC GCC AGG GCA ATG AAT	CTG GAT GAT GTA GAC GCC CC
*Gabrg2* (*WT*)	AGT TCG CCA AAT ACA TGG AGC	GTA GAG CGA TAG CAG GAG CA
*Gabrg2* (*Cre excision*)	ACC ATG ACA ACT TTA AGC ACC A	CTT GCT TGG CTT CCG GTT G

### Electrophysiology

Adult *Gabrd* and *Gabrg2* mice were bilaterally injected with either AAV-GFP or AAV-Cre-GFP and allowed to recover for three weeks prior to electrophysiological recording. Tonic and phasic GABAergic currents were measured using whole-cell voltage clamp recording techniques as previously described in visually-identified GFP expressing neurons in the VTA (Lee & Maguire 2013). Briefly, mice were anesthetized with isoflurane and decapitated, and the brain was rapidly removed and placed immediately in ice-cold, oxygenated normal artificial cerebrospinal fluid [nACSF; containing (in mM) 126 NaCl, 26 NaHCO_3_, 1.25 NaH_2_PO_4_, 2.5 KCl, 2 CaCl_2_, 2 MgCl_2_, and 10 dextrose (300–310 mOsm)] containing 3 mM kynurenic acid and bubbled with 95% O_2_–5% CO_2_. Coronal slices (350 mm thick) containing the VTA were prepared using a Leica VT1000S vibratome. For voltage-clamp recordings, slices were maintained in oxygenated nACSF containing 3 mM kynurenic acid. For current-clamp recordings, slices were maintained in oxygenated nACSF. After a 1 h minimum recovery period, VTA-containing slices were placed into a recording chamber maintained at 33 °C (in-line heater; Warner Instruments) and perfused at a high flow rate (4 ml/min) throughout the experiment. SR95531 (200 μM) was added to the extracellular solution where indicated.

For voltage-clamp recordings, the intracellular recording solution contained (in mM) 140 CsCl, 1 MgCl_2_, 10 HEPES, 4 NaCl, 0.1 EGTA, 2 Mg-ATP, and 0.3 Na-GTP (pH 7.25, 280–290 mOsm). Electrodes were used with DC resistance of 5–8 MOhm. Following stabilization of the holding current and series resistance and capacitance measurements, spontaneous inhibitory postsynaptic currents (sIPSCs) were recorded in GFP+ cells from both AAV-GFP and AAV-Cre-GFP slices over a five-minute period at a holding potential of −70 mV. The frequency, peak amplitude, and weighted decay (τ_w_) of sIPSCs were measured using MiniAnalysis software (version 6.0.3, Synaptosoft Inc., Fort Lee, NJ, USA). Tonic GABAergic currents were measured as previously described^22,23^ in visually identified GFP+ cells from both AAV-GFP and AAV-Cre-GFP slices. The mean current was measured during 10 ms epochs collected every 100 ms throughout the experiment. A Gaussian was fit to points over a 60 s period before and after bath application of SR95531 (>200 µM) to determine the mean holding current in nACSF and in the presence of SR95531. The difference in the holding current in the presence or absence of SR95531 was determined to be a measure of tonic GABAergic inhibition. Series resistance and whole cell capacitance were continually monitored and compensated throughout the course of the experiment. Recordings were eliminated from data analysis if series resistance increased by 20%.

### Statistical analyses

All statistical analyses were conducted using Prism 7 (GraphPad La Jolla, CA, USA). Repeated measures, two-way ANOVA was used to analyze DID 2 h drinking data, with AAV (GFP versus Cre) as the between-subjects factor and time as the within-subjects factor. Repeated measures, two-way ANOVAs were used to analyze both the sucrose preference and quinine aversion experiments, with AAV as the between-subjects factor and time as the within-subjects factor. An unpaired, two-tailed Student’s t test was used to analyze DID binge, 4 h drinking data (GFP versus Cre). Unpaired, two-tailed Student’s t tests were also used to analyze baseline and Cre-excision qPCR data (male versus female, GFP versus Cre, respectively). A Pearson’s correlation was used to analyze the relationship between AAV transduction (optical density) and binge drinking as well as intake (g/kg) and BECs. Data are presented as the average ± s.e.m. A p-value of p < 0.05 was considered statistically significant for all analyses.

## Results

### Sex differences in *Gabrd* mRNA expression in the VTA

**Figure 1:**
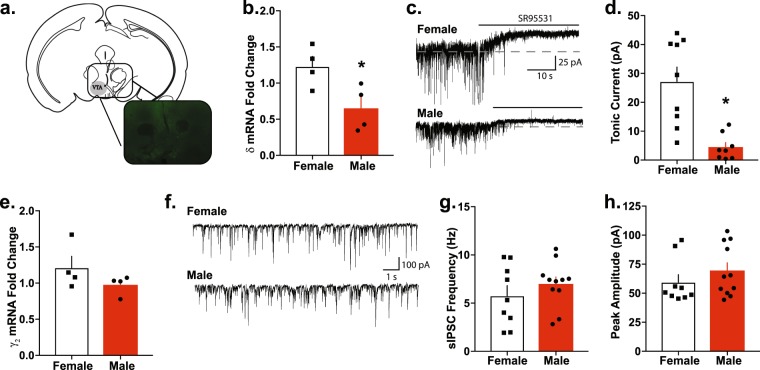
Higher Gabrd transcript levels and tonic inhibition in the VTA of female mice. (**a**) Bilateral VTA punches were obtained from C57Bl/6J male and female mice, as indicated in the diagram adapted from^47^ and shown in the representative image (40 µm). (**b**) qPCR analysis revealed higher Gabrd transcript levels in female VTA relative to males. (*p = 0.032, n = 4 per group). A separate cohort of mice received bilateral, AAV-GFP injections into the VTA and were assessed for baseline differences in tonic current three weeks post-op. Representative trace (**c**) illustrating the effect on tonic current after application of the GABA_A_R antagonist Gabazine in male and female mice. (**d**) Addition of Gabazine resulted in a larger tonic current in female VTA relative to male. (p < 0.01) (**e**) There was no effect of sex on *Gabrg2* transcript levels in the VTA (p = 0.203). (**f**–**h**) There were no significant functional differences in phasic inhibition between male and female VTA, as indicated by the lack of difference in both sIPSC frequency and peak amplitude (p = 0.3304 and p = 0.3075, respectively; n = 9–10 cells, 3 mice per experimental group).

Total mRNA was isolated from tissue punches from the VTA of males and females (Fig. 1a). The ratio of *Gabrd* mRNA to β-actin levels were higher in the VTA of females (females 1.23 ± 0.14 fold) compared to males (0.65 ± 0.16 fold) (t(6) = 2.78, p = 0.032, Fig. 1b). Consistent with higher *Gabrd* transcript levels in females, tonic inhibitory currents were higher in neurons in the VTA of females (27.23 ± 4.96 pA) relative to males (8.55 ± 4.29 pA) (t(16) = 2.85, p = 0.012, Fig. 1c,d). Collectively, these results show a greater δ-mediated, tonic inhibition in the VTA of female mice relative to males.

### No sex differences in VTA *Gabrg2* mRNA *expression*

We next sought to determine whether these effects were specific to δ-mediated tonic inhibition or if they applied to GABAergic inhibition more broadly. First, we determined that there are no sex differences in ratio of *Gabrg2* transcript levels compared to β-actin in the VTA between males (0.98 ± 0.07 fold) and females (1.21 ± 0.16 fold) (t(6) = 1.384, p = 0.2156, Fig. 1e) and there was no significant difference in frequency of sIPSCs in VTA neurons between females (5.77 ± 1.05 Hz) and males (frequency: 7.00 ± 0.71 Hz) (t(18) = 1, p = 0.3304, Fig. 1f,g). There is also no difference in the peak amplitude of sIPSCs in VTA neurons between females (59.54 ± 6.49 pA) and males (69.49 ± 6.72 pA) (t(18) = 1.05, p = 0.3075, Fig. 1f,h). Taken together, these results indicate that there are similar levels of *Gabrg2* transcript and ɣ2-mediated phasic inhibition in the VTA of males and females.

### Knockdown of *Gabrd* in the VTA

Given the baseline differences in δ-mediated tonic inhibition in the VTA between males and females (Fig. 1), it is likely that excision of the *Gabrd* gene would have a greater impact on females. As such, we next sought to determine whether excision of the *Gabrd* gene would result in differential changes in VTA tonic inhibition between males and females.

**Figure 2:**
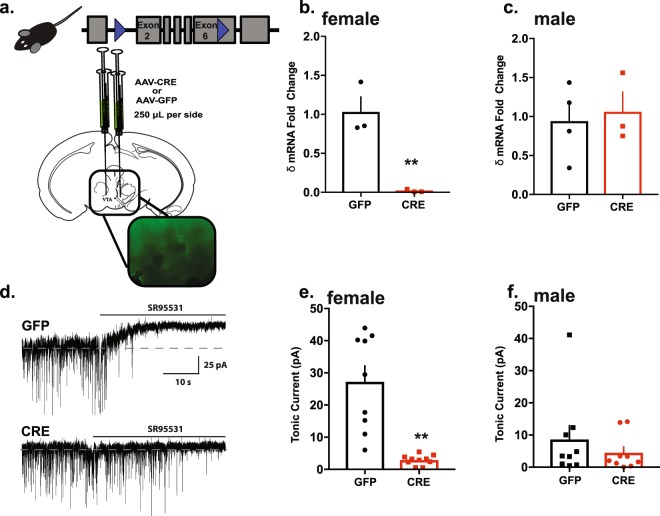
Cre-mediated Gabrd knockdown in VTA results in decreased Gabrd transcript levels and reduced tonic inhibition only in females. (**a**) Floxed Gabrd mice were generated to allow for excision of exons 2 through 6b of the Gabrd gene. Bilateral 0.5 mm VTA punches were obtained from floxed Gabrd male and female mice bilaterally injected with either AAV-GFP or AAV-Cre-GFP as shown in the diagram adapted from^47^. (**b**) qPCR analysis revealed a significant decrease in Gabrd transcript in the VTA of female AAV-Cre-GFP (n = 3) relative to AAV-GFP (n = 3) controls (**p = 0.006). (**c**) qPCR analysis revealed no significant difference between male AAV-Cre-GFP (n = 3) and AAV-GFP (n = 4) controls. Significant decrease in tonic inhibition was seen between female AAV-Cre-GFP and AAV-GFP (*p < 0.0001). (**d**) Representative traces of the tonic current recorded from the VTA of female AAV-GFP (top) and female AAV-Cre-GFP (bottom). (**e**) Change in holding current after application of Gabazine is nearly absent in slices obtained from females with Cre-mediated excision of Gabrd, indicating near ablation of δ-mediated tonic inhibition. (**f**) No change in tonic inhibition was found between male AAV-Cre-GFP and AAV-GFP groups (n = 9–10 cells, 3 mice per experimental group).

Total mRNA was isolated from tissue punches from the VTA of male and female mice stereotaxically injected with either AAV-GFP or AAV-Cre-GFP (Fig. 2a). *Gabrd* mRNA levels compared to β-actin were reduced in in the VTA of AAV-Cre-GFP-injected females (0.02 ± 0.01 fold) relative to female AAV-GFP controls (1.03 ± 0.19 fold) (t(4) = 5.28, p = 0.006, Fig. 2b). In contrast, there was no significant difference in the ratio of *Gabrd* to β-actin mRNA levels between male AAV-Cre-GFP (1.06 ± 0.25 fold change) and male AAV-GFP mice (0.94 ± 0.24 fold change) (t(5) = 0.34, p = 0.75, Fig. 2c).

Consistent with the expression data, we observed a significant decrease in tonic current in the VTA of female AAV-Cre-GFP (2.90 ± 0.49 pA) compared to female AAV-GFP controls (27.23 ± 4.96 pA) (t(17) = 5.16, p < 0.0001, Fig. 2d,e). In contrast, there was no difference between in the tonic current measured in VTA neurons from male AAV-Cre-GFP (4.46 ± 1.85 pA) or male AAV-GFP mice controls (8.55 ± 4.29 pA) (t(16) = 0.88, p = 0.394, Fig. 2d,f).

Collectively, these results indicate that Cre-mediated excision of the Gabrd gene can effectively decrease both *Gabrd* mRNA transcript levels and tonic inhibition in the VTA. While this effect was only notable in females, its apparent ineffectiveness in males is likely due to the floor effect of low baseline *Gabrd* expression.

### Knockdown of *Gabrd* in the VTA results in sex-specific differences in binge-like drinking

**Figure 3:**
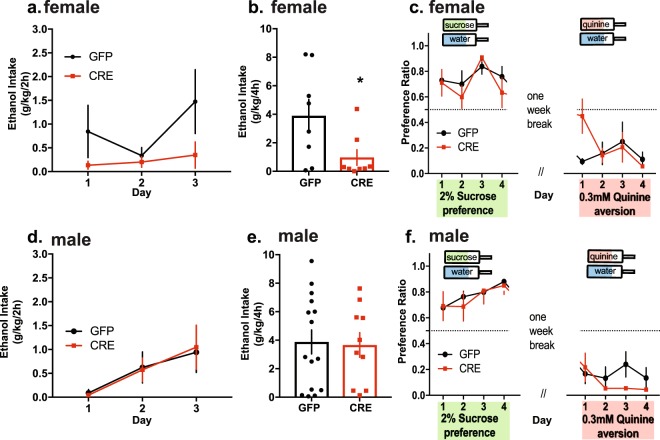
Gabrd excision from the VTA selectively reduces female binge-like drinking. Gabrd excision resulted in a significant difference in female binge-like drinking with no change in males. (**a**) VTA Gabrd excision had no effect on female 20% alcohol drinking across the first three days of 2 h access, but (**b**) led to a significant decrease in binge-like drinking (*p = 0.037). (**c**) In contrast, there were no main effect of experimental group nor interaction of experimental group and day on sucrose preference or quinine preference for females. (**d**) VTA Gabrd excision had no effect on male 20% alcohol drinking over the first three days of 2 h access and (**e**) no effect on binge-like, 4 h, drinking. Male and female AAV-Cre-GFP and AAV-GFP mice were subjected to 4 d of sucrose (2%, w/v) preference. After a 3 d break, they were then subjected to 4 d of 0.3 mM quinine aversion. (**f**) There were no main effect of experimental group nor interaction of experimental group and day on sucrose preference or quinine preference for males.

Given the putative role for δ subunit-containing GABA_A_ receptors in mediating the effects of low dose alcohol, we next sought to determine whether δ subunit-containing GABA_A_ receptors had a sex-dependent effect on binge drinking. To address this question, we implemented the Drinking in the Dark paradigm (DID^28^). During the first three days, mice were allowed 2 h of access to 20% (v/v) ethanol that was timed to occur three hours after the start of the dark cycle. There was no interaction between *Gabrd* mRNA knockdown and alcohol drinking for the first three days for either females or males (females: F(2,28) = 1.93, p = 0.16; males: F(2,46) = 0.07, p = 0.93) (Fig. 3a,d). The fourth and final day was the ‘binge’ day, where animals had access to alcohol for 4 h. Females in the intra-VTA AAV-Cre-GFP group consumed significantly less alcohol during the binge day (0.97 ± 0.55 g/kg) compared to female intra-VTA AAV-GFP controls (3.90 ± 1.14 g/kg) (t(14) = 2.31, p = 0.037, Fig. 3b). Males, however, showed no difference in binge-like drinking between AAV-Cre-GFP (3.67 ± 0.87 g/kg) and AAV-GFP groups (3.88 ± 0.84 g/kg) (t(23) = 0.17, p = 0.868, Fig. 3e).

To validate g/kg intake values against measurable BECs, a subset of the male and female mice subjected to DID had submandibular blood taken immediately after the 4 h ‘binge’ session. There was a significant correlation across subjects and AAV group between BEC and g/kg intake (r = 0.976, n = 12, p < 0.0001, data not shown), verifying that g/kg values were an accurate reflection of g/kg alcohol consumed.

To determine if these effects were specific for alcohol, sucrose preference and quinine aversion was assessed. There were no significant differences between female AAV-Cre-GFP or AAV-GFP groups (Fig. 3c) or male AAV-Cre-GFP or AAV-GFP groups (Fig. 3f) in their preference for 2% (w/v) sucrose (females: F(3,42) = 0.62, p = 0.61; males: F(3,54) = 0.19, p = 0.90) or their aversion to 0.3 mM quinine (females: F(3,42) = 2.57, p = 0.07; males: F(3,51) = 1.08, p = 0.37).

### Knockdown of *Gabrd* in the pVTA of females is correlated with decreased binge-like drinking

**Figure 4:**
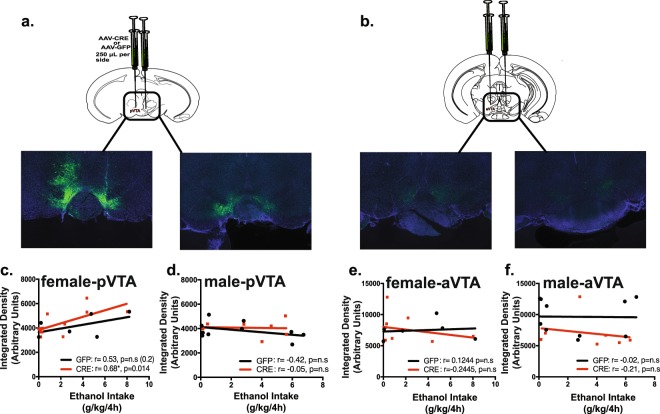
Significant correlation between female AAV-Cre-GFP transduction efficiency in the pVTA of floxed Gabrd mice and binge-like drinking. Both aVTA and pVTA slices from floxed Gabrd mice were selected for placement analysis and were spread throughout the rostrocaudal axis (−2.8 mm to −3.88 mm, A/P, relative to bregma) as depicted in the diagrams adapted from^47^ (**a**,**b**). (**a**) Significantly higher levels of GFP reporter expression were noted in the pVTA of both AAV-Cre-GFP and AAV-GFP mice, indicating higher AAV transduction in this region compared to the aVTA (**b**). (**c**) There was a significant correlation between binge-like drinking and AAV transduction in the pVTA of female AAV-Cre-GFP (r = 0.68, p = 0.014), but not AAV-GFP control females (r = −0.253, p = 0.2). (**d**) There was no significant correlation between intake and AAV transduction in the pVTA for either AAV-CRE or GFP males There was no significant correlation between intake and AAV transduction in the aVTA for either AAV-CRE or GFP females (**e**). (**f**) Similarly, there was no significant correlation between intake and AAV transduction in the aVTA for either AAV-Cre-GFP or AAV-GFP males (n = 7–10 mice per group). N.B. Photomicrographs were analyzed as binary masks, thus Integrated density values experienced inversion of pixilation and low density values indicate higher levels of GFP reporter expression.

Based on the well-established heterogeneity of the VTA with DA and GABAergic neurons being differentially distributed along several dimensions including the rostrocaudal axis (Olson *et al*. 2005; Ikemoto 2007; Chieng *et al*. 2011) and differences in responses to GABAergic agonists and antagonists (Ikemoto, Murphy & McBride. 1998; Ikemoto 2010; Melón *et al*. 2017), we next examined whether there was a neuroanatomical relationship within the VTA between AAV transduction efficiency and binge-like drinking in females and males. An initial placement analysis revealed that both AAV-Cre-GFP and AAV-GFP transduction were predominantly targeted to the posterior VTA (pVTA) (Fig. 4a), with little viral spread to the anterior VTA (aVTA) (Fig. 4b).

There was a significant correlation between pVTA viral transduction in female AAV-Cre-GFP mice and binge-like drinking (r = 0.683, p = 0.014, Fig. 4c), but not between pVTA viral transduction in female AAV-GFP mice (r = 0.535, p = 0.274; Fig. 4c). In contrast, there was no significant relationship between pVTA viral transduction in either male AAV-Cre-GFP (r = −0.051, p = 0.914) or male AAV-GFP (r = −0.419, r = 0.229) and binge-like drinking (Fig. 4d).

There was no significant relationship in the viral transduction in the aVTA of female AAV-Cre-GFP (r = −0.245, p = 0.526) or female AAV-GFP mice and binge-like drinking (r = 0.124, p = 0.814) (Fig. 4e). Similarly, there was also no significant relationship between aVTA viral transduction in either male AAV-Cre-GFP (r = −0.212, r = 0.648) or male AAV-GFP (r = −0.0167, r = 0.963) mice and binge-like drinking (Fig. 4f).

Analysis of relevant spillover areas (e.g. SNr, IPN) also revealed no significant correlations with binge-like drinking in males or females (female AAV-GFP SNr: r = 0.0494, p = 0.91; female AAV-Cre-GFP SNr: r = 0.1396, p = 0.67; male AAV-GFP SNr: r = −0.06, p = 0.92; male AAV-Cre-GFP SNr: r = 0.94, p = 0.88) (female AAV-GFP IPN: r = −0.047, p = 0.93; female AAV-Cre-GFP IPN: r = −0.21, p = 0.51; male AAV-GFP IPN: r = −0.031, p = 0.93; male AAV-Cre-GFP IPN: r = −0.181, p = 0.70) (data not shown). Although correlative, these results indicate a significant relationship between efficiency of viral transduction in the pVTA of females and later binge-like drinking.

### Reduction in *Gabrg2* expression in the VTA

**Figure 5:**
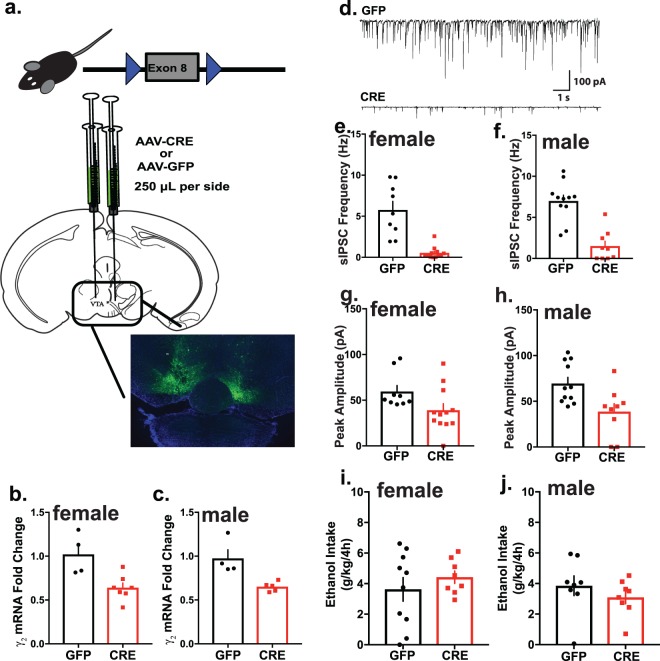
Cre-mediated *Gabrg2* excision in VTA results in decreased *Gabrg2* transcript levels and phasic inhibition. (**a**) Floxed *Gabrg2* mice were used to excise exon 8 of the *Gabrg2* gene as previously described^47^ via stereotaxic injection of AAV-Cre-GFP into the VTA as shown in the diagram adapted from^48^. *Gabrg2* transcript levels were significantly decreased in female (**b**) and male (**c**) AAV-Cre-GFP VTA relative to AAV-GFP (n = 4–7 mice per group). (**d**) Representative trace demonstrating decreased phasic inhibition after Cre-mediated excision of *Gabrg2*. Cre-mediated excision resulted in decreases in both sIPSC frequency and peak amplitude in GFP+ cells from the VTA of female (**e**,**g**) and male (**f**,**h**) AAV-Cre-GFP relative to AAV-GFP (***p < 0.0001, ^^^p = 0.053, ***p < 0.0001 and **p = 0.01; n = 9–12 cells, 3 mice per experimental group). VTA *Gabrg2* excision had no effect on binge-like drinking in females (**i**) or males (**j**).

To examine whether the sex-dependent effects on binge drinking were unique to knockdown of the *Gabrd* gene or apply more broadly to alterations in GABAergic signaling, adult male and female floxed *Gabrg2* mice were bilaterally injected with either AAV-Cre-GFP or AAV-GFP into the VTA (Fig. 5a). There was significant decrease in *Gabrg2* transcript levels in female AAV-Cre-GFP (0.64 ± 0.054 fold) relative to female AAV-GFP (1.02 ± 0.12 fold) (t(9) = 3.37, p = 0.008, Fig. 5b). Similarly, male AAV-Cre-GFP VTA *Gabrg2* levels (0.65 ± 0.02 fold) were significantly lower when compared to *Gabrg2* transcript levels from male AAV-GFP controls (0.97 ± 0.1 fold) (t(7) = 3.53, p = 0.009, Fig. 5c).

The frequency (0.54 ± 0.20 Hz) and peak amplitude (39.19 ± 6.98 pA) of sIPSCs were decreased in female AAV-Cre-GFP relative to female AAV-GFP (frequency: 5.77 ± 1.05 Hz; peak amplitude: 59.54 ± 6.49 pA, t(19) = 5.63, p < 0.0001 and t(19) = 2.068, p = 0.0526, respectively, Fig. 5d,e,g). Similarly, the frequency (1.50 ± 0.61 Hz) and peak amplitude (38.73 ± 8.75 pA) of sIPSCs was decreased in male AAV-Cre-GFP relative to AAV-GFP (7.00 ± 0.71 Hz; peak amplitude: 69.49 ± 6.72 pA) (t(18) = 5.735, p < 0.0001 and t(18) = 2.836, p = 0.0110, respectively, Fig. 4d,f,h). Collectively, these results reveal that Cre-mediated excision decreased *Gabrg2* transcript levels as well as reduced phasic GABAergic inhibition in the VTA of both males and females.

### Reduced VTA *Gabrg2* expression does not alter binge-like drinking

Floxed *Gabrg2* male and female mice were bilaterally injected into the VTA with either AAV-Cre-GFP or AAV-GFP and subjected to one cycle of binge-like drinking (DID) as previously described. There was no significant difference in binge-like drinking in AAV-Cre-GFP or AAV-GFP females (3.63 ± 0.77 g/kg, 4.42 ± 0.4 g/kg, respectively; p = 0.41, Fig. 5i). Similarly, there is no difference in binge-like drinking in AAV-Cre-GFP and AAV-GFP males (3.09 ± 0.42 g/kg, 3.85 ± 0.64 g/kg, respectively; p = 0.34, Fig. 5j). There was a significant correlation between BEC measures and g/kg intake (r = 0.5786, n = 26, p = 0.002; data not shown), validating the use of g/kg as a drinking measure.

There were also no significant differences in sucrose preference (females: F(3,45) = 1.01, p = 0.40; males: F(3,54) = 0.19, p = 0.90) or quinine aversion (females: F(3,45) = 0.02, p = 1.00; males: F(3,51) = 1.08, p = 0.37) in males or females as a result of *Gabrg2* excision from the VTA (data not shown). These results indicate that the γ2 subunit in the VTA does not impact binge-like drinking.

## Discussion

The results presented here show a striking and novel sex-specific difference in murine binge-like alcohol drinking that hinges on *Gabrd* expression in the pVTA. Concurrently, we also show higher baseline *Gabrd* transcript levels as well as δ-mediated tonic inhibition in the VTA of female mice as compared to males. In contrast, sex-specific differences were not observed in transcript levels of *Gabrg2* or in phasic GABAergic inhibition. Subsequent Cre-mediated *Gabrd* excision primarily from the pVTA significantly reduced female binge-like drinking, with Cre-mediated *Gabrg2* excision having no effect. Taken together, these results indicate that δ-mediated tonic inhibition in the VTA plays an integral role in mediating binge drinking behavior in females.

Recent work has demonstrated estrous cycle-dependent changes in *Gabrd* transcript levels in the VTA^24^. Specifically, *Gabrd* levels in non-estrus females were found to be approximately 80% greater than those in estrus. More broadly, this pattern underscores that ovarian hormones and, relatedly, biological sex plays a relevant role in regulating *Gabrd* expression. The female mice used in these experiments were maintained in same-sex housed conditions under separate ventilation from males, thus avoiding estrus cycle-inducing male cues^26,27^, focusing the study solely on sex-dependent effects on binge drinking. The highest relative *Gabrd* female transcript level was approximately 50% greater than our lowest relative *Gabrd* transcript level in acyclic females, which is markedly less variable than that seen between estrous and non-estrous states in freely cycling females^24^, indicating a likely less important role for estrous cycle in these acyclic females. Perhaps most importantly, Cre-mediated excision reduced VTA *Gabrd* levels in females to nearly negligible levels. Thus, any cycling-dependent effects on subunit expression for *Gabrd* mice would be irrelevant once Cre-mediated excision had occurred.

While there have been conflicting reports^29^, it has been suggested that low alcohol concentrations influence the activity of δ-containing GABA_A_ receptors in recombinant receptor systems as well as in slices^9–11,30,31^. However, it is also likely that the well-documented effects of ethanol on presynaptic GABA release^15–17^ and/or increases in neurosteroid levels^18–20,32^ may indirectly mediate the effects of ethanol on these receptors. Regardless, global deletion of the *Gabrd* gene was found to be associated with reduced 24 hr alcohol consumption and preference in males and females^12^. Yet, whether similar sex-independent reductions in intake would occur when modeling binge-like drinking had not been determined. The present work allowed for site-specific *Gabrd* deletion within the VTA, demonstrating a sex-dependent role of GABA_A_R δ subunit-containing receptors in binge-like alcohol consumption. It is also possible that *Gabrd* excision in other areas of mesolimbic circuitry (e.g., dorsomedial shell of the nucleus accumbens, see^33^) would be more effective in males and/or could occlude the bias conferred by the δ in the VTA of females.

When compared with δ-containing GABA_A_ receptors, γ2-containing GABA_A_ receptors are extensively expressed throughout the brain^34^. Clinically, there is some evidence that alterations to *Gabrg2* is related to the propensity to develop alcoholism^35–37^ and past work has shown γ2-subunit containing receptors respond to high concentrations of alcohol (>30–40 mM^31^). This indicates that γ2-containing GABA_A_ receptors are less sensitive to alcohol—especially relative to those with a δ subunit. When considering our *Gabrg2* mice, it is notable that the level of reduction in *Gabrg2* levels was not as significant as those seen in Cre-mediated excision of *Gabrd*. One concern is that the remaining γ2 subunit-containing GABA_A_ receptors might counterbalance any effect of the knockdown. Our *Gabrg2* mice drank an average of 3.5–4.5 g/kg for females (Cre and GFP) and 3.0–3.8 g/kg for males (Cre and GFP), corresponding to approximate BECs of 40 mg/dL immediately after the ‘binge’. These values are lower than have been reported for DID^7,28,38^, and approximate to alcohol concentrations of 8–9 mM. This is potentially lower than the threshold for γ2 subunit activation, which may impact our results and introduce a bias towards δ-mediated effects.

It is worth noting that the DID protocol requires single housing of the animals, introducing a social isolation stress using this paradigm, which may impact alcohol consumption^39^. In particular, social isolation during adolescence has been shown to impact alcohol consumption in adulthood^40,41^ in a sex-specific manner^42^. In the current study, the animals are group housed until being subjected to the DID protocol as adults, suggesting that the differences in alcohol consumption is not related to previous social isolation stress. In addition, all the animals in the study are identically treated and subjected to the same extent of social isolation, arguing that differences in intake following genetic manipulations or the sex-dependent differences are not related to the social isolation component of the experimental paradigm.

**Figure 6:**
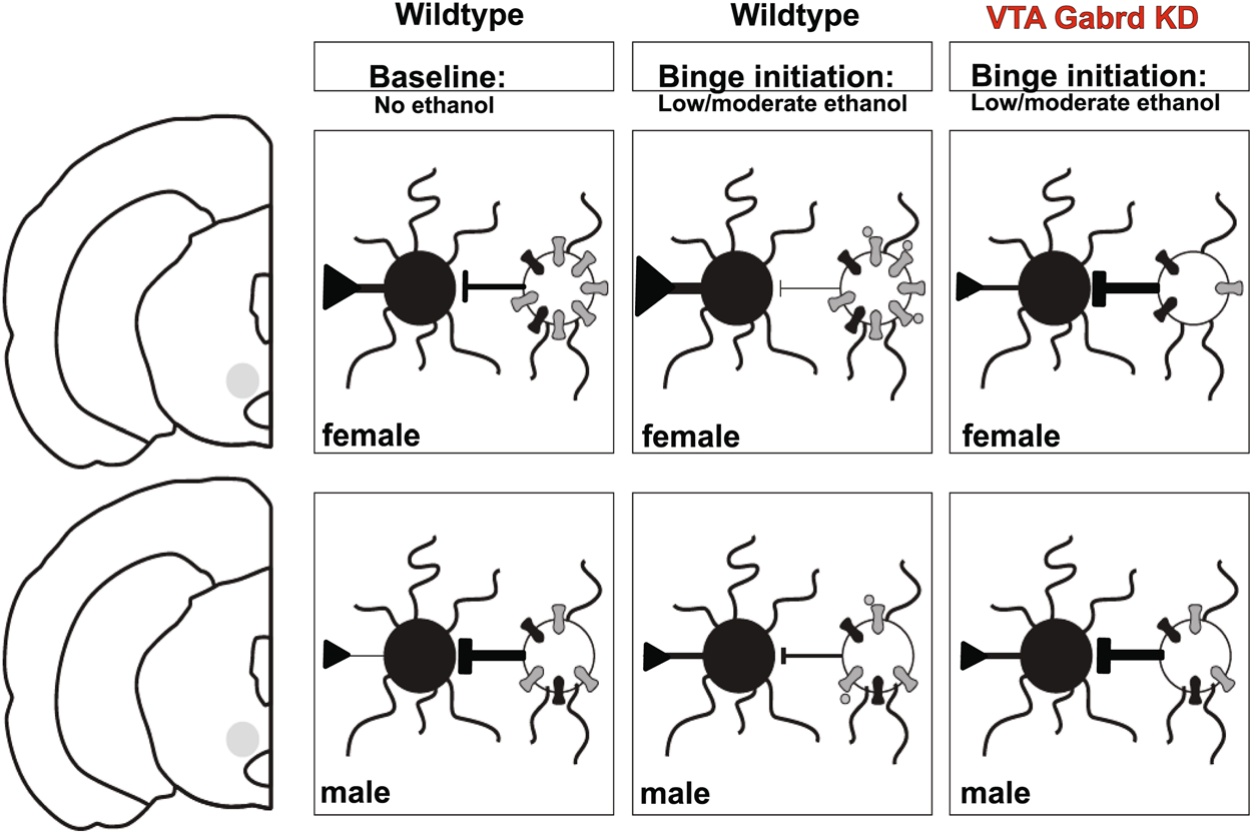
Higher δ expression in the VTA of females has implications for overall mesolimbic excitability and effects on binge drinking. (top row, left to right) Female VTA has higher expression of δ-subunit containing GABA_A_Rs in the VTA, resulting in lower inhibition on DAergic neurons and higher DA release. Low concentrations of alcohol interact with δ-subunit containing GABA_A_Rs on this population, further increasing DA release. Cre-mediated excision markedly reduces Gabrd expression and δ-mediated tonic inhibition, reducing overall DA release in females. (bottom row, left to right) Male VTA has decreased baseline δ-subunit containing GABAA receptor in the VTA, resulting in greater inhibition on DAergic projection neurons. While low concentrations of alcohol disinhibit DAergic neurons and increase DA release, the effect is not as significant as in females. Due to the low baseline Gabrd levels, Cre-mediated excision has limited effect on both Gabrd levels, δ-mediated tonic inhibition, and resulting DA release. In all cases, γ2-containing GABAA receptors are present at equal levels between males and females and are not affected by low concentrations of alcohol.

Our data revealed two major conclusions: (1) higher baseline *Gabrd* levels in females and (2) reductions in binge-like drinking after *Gabrd* excision from the VTA in females. But what does this mean for overall differences in VTA circuit functioning between males and females? In a baseline state, female mice have a larger amount of δ-mediated tonic inhibition within the VTA, whether localized exclusively to the pVTA or more broadly. As previous work supports greater activation of GABAergic vs. DAergic cells in the VTA in response to the δ-specific compound THIP^43^, we posit that the sex difference in δ-GABA_A_Rs would maintain higher inhibitory tone on these GABAergic cells, allowing for the greater activation of dopamine neurons in the VTA in females^25^ (Fig. 6) and sex/estrous differences in striatal dopamine release from these VTA originating DA projection^25,44^. After binge-like drinking, low amounts of alcohol augment activity of δ subunit-containing GABA_A_ receptors, thereby enhancing tonic inhibition on GABAergic cells. This further disinhibition of local GABAergic populations could allow for even greater DA release in the NAc (Fig. 6). Consistent with this interpretation, female Wistar rats trained to lever press for alcohol were shown to have greater DA release after low dose (0.25 and 0.5 g/kg) alcohol administration relative to males^45^. Removal of *Gabrd* from this population of GABAergic interneurons through Cre-mediated excision would not only reduce baseline tonic inhibition but would also eliminate a key allosteric mechanism for low-dose alcohol effects in this region of the midbrain in females, reducing the mechanism driving binge drinking behavior.

Collectively, this work demonstrates the importance of δ subunit-containing GABA_A_ receptors in the VTA of female mice during binge-like drinking. Further, the evidence of a role for δ subunit-containing GABA_A_ receptors in alcohol consumption also has implications regarding withdrawal, in which changes in δ expression have been observed in the hippocampus^46^; however, it remains to be determined whether alcohol exposure alters GABA_A_R δ subunit expression in brain regions implicated in alcohol intake, such as the VTA.

## References

[1] NIAAA, N. I. o. A. A. a. A. NIAAA council approves definition of binge drinking. *NIAAA newsletter* **3** (2004).

[2] Naimi TS (2003). Binge drinking among US adults. JAMA.

[3] White A (2015). Converging Patterns of Alcohol Use and Related Outcomes Among Females and Males in the United States, 2002 to 2012. Alcohol Clin Exp Res.

[4] Zilberman M, Tavares H, el-Guebaly N (2003). Gender similarities and differences: the prevalence and course of alcohol- and other substance-related disorders. J Addict Dis.

[5] Hwa LS (2011). Persistent escalation of alcohol drinking in C57BL/6J mice with intermittent access to 20% ethanol. Alcohol Clin Exp Res.

[6] Melón LC, Wray KN, Moore EM, Boehm SL (2013). Sex and age differences in heavy binge drinking and its effects on alcohol responsivity following abstinence. Pharmacol Biochem Behav.

[7] Rhodes JS (2007). Mouse inbred strain differences in ethanol drinking to intoxication. Genes Brain Behav.

[8] Mehta AK, Ticku MK (1988). Ethanol potentiation of GABAergic transmission in cultured spinal cord neurons involves gamma-aminobutyric acidA-gated chloride channels. J Pharmacol Exp Ther.

[9] Olsen RW, Hanchar HJ, Meera P, Wallner M (2007). GABAA receptor subtypes: the “one glass of wine” receptors. Alcohol.

[10] Wallner M, Hanchar HJ, Olsen RW (2006). Low dose acute alcohol effects on GABA A receptor subtypes. Pharmacol Ther.

[11] Glykys J (2007). A new naturally occurring GABA(A) receptor subunit partnership with high sensitivity to ethanol. Nat Neurosci.

[12] Mihalek RM (2001). GABA(A)-receptor delta subunit knockout mice have multiple defects in behavioral responses to ethanol. Alcohol Clin Exp Res.

[13] Wei W, Faria LC, Mody I (2004). Low ethanol concentrations selectively augment the tonic inhibition mediated by delta subunit-containing GABAA receptors in hippocampal neurons. J Neurosci.

[14] Santhakumar V, Wallner M, Otis TS (2007). Ethanol acts directly on extrasynaptic subtypes of GABA(A) receptors to increase tonic inhibition. Alcohol (Fayetteville, N.Y.).

[15] Lovinger David M. (2017). Presynaptic Ethanol Actions: Potential Roles in Ethanol Seeking. The Neuropharmacology of Alcohol.

[16] Melis M, Camarini R, Ungless MA, Bonci A (2002). Long-lasting potentiation of GABAergic synapses in dopamine neurons after a single *in vivo* ethanol exposure. Journal of Neuroscience.

[17] Roberto M, Madamba SG, Moore SD, Tallent MK, Siggins GR (2003). Ethanol increases GABAergic transmission at both pre- and postsynaptic sites in rat central amygdala neurons. Proceedings of the National Academy of Sciences.

[18] Sanna E (2004). Brain steroidogenesis mediates ethanol modulation of GABAA receptor activity in rat hippocampus. Journal of Neuroscience.

[19] Barbaccia ML (1999). Ethanol markedly increases “GABAergic” neurosteroids in alcohol-preferring rats. European Journal of Pharmacology.

[20] Beattie, M. C., Maldonado-Devincci, A., Cook, J. B. & Morrow, A. L. In *Neuropathology of Drug Addictions and Substance Misuse* 433–444 (Elsevier, 2016).

[21] Belelli D, Lambert JJ (2005). Neurosteroids: endogenous regulators of the GABA A receptor. Nature Reviews Neuroscience.

[22] Maguire JL, Stell BM, Rafizadeh M, Mody I (2005). Ovarian cycle-linked changes in GABA(A) receptors mediating tonic inhibition alter seizure susceptibility and anxiety. Nat Neurosci.

[23] Maguire J, Mody I (2007). Neurosteroid synthesis-mediated regulation of GABA(A) receptors: relevance to the ovarian cycle and stress. J Neurosci.

[24] Melón LC, Nolan ZT, Colar D, Moore EM, Boehm SL (2017). Activation of extrasynaptic delta-GABAA receptors globally or within the posterior-VTA has estrous-dependent effects on consumption of alcohol and estrous-independent effects on locomotion. Horm Behav.

[25] Calipari ES (2017). Dopaminergic dynamics underlying sex-specific cocaine reward. Nat Commun.

[26] Tyl, R. W. Guidelines for mating rodents. *Curr Protoc Toxicol* Chapter 16, Unit 16 12, 10.1002/0471140856.tx1602s11 (2002). 10.1002/0471140856.tx1602s11 23045078

[27] Whitten WK (1956). Modification of the oestrous cycle of the mouse by external stimuli associated with the male. J Endocrinol.

[28] Rhodes JS, Best K, Belknap JK, Finn DA, Crabbe JC (2005). Evaluation of a simple model of ethanol drinking to intoxication in C57BL/6J mice. Physiol Behav.

[29] Borghese CM (2006). The delta subunit of gamma-aminobutyric acid type A receptors does not confer sensitivity to low concentrations of ethanol. J Pharmacol Exp Ther.

[30] Hanchar HJ, Dodson PD, Olsen RW, Otis TS, Wallner M (2005). Alcohol-induced motor impairment caused by increased extrasynaptic GABA(A) receptor activity. Nat Neurosci.

[31] Wallner M, Hanchar HJ, Olsen RW (2003). Ethanol enhances alpha 4 beta 3 delta and alpha 6 beta 3 delta gamma-aminobutyric acid type A receptors at low concentrations known to affect humans. Proc Natl Acad Sci USA.

[32] Morrow AL, VanDoren MJ, Fleming R, Penland S (2001). Ethanol and neurosteroid interactions in the brain. Int Rev Neurobiol.

[33] Nie H, Rewal M, Gill TM, Ron D, Janak PH (2011). Extrasynaptic delta-containing GABAA receptors in the nucleus accumbens dorsomedial shell contribute to alcohol intake. Proc Natl Acad Sci USA.

[34] Pirker S, Schwarzer C, Wieselthaler A, Sieghart W, Sperk G (2000). GABA(A) receptors: immunocytochemical distribution of 13 subunits in the adult rat brain. Neuroscience.

[35] Koulentaki Mairi, Kouroumalis Elias (2018). GABAA receptor polymorphisms in alcohol use disorder in the GWAS era. Psychopharmacology.

[36] Li D (2014). Association of gamma-aminobutyric acid A receptor alpha2 gene (GABRA2) with alcohol use disorder. Neuropsychopharmacology.

[37] Buck KJ, Hood HM (1998). Genetic association of a GABA(A) receptor gamma2 subunit variant with severity of acute physiological dependence on alcohol. Mamm Genome.

[38] Newman EL (2016). Effects of Gabra2 Point Mutations on Alcohol Intake: Increased Binge-Like and Blunted Chronic Drinking by Mice. Alcohol Clin Exp Res.

[39] Doremus TL, Brunell SC, Rajendran P, Spear LP (2005). Factors influencing elevated ethanol consumption in adolescent relative to adult rats. Alcohol Clin Exp Res.

[40] Chappell AM, Carter E, McCool BA, Weiner JL (2013). Adolescent rearing conditions influence the relationship between initial anxiety-like behavior and ethanol drinking in male Long Evans rats. Alcohol Clin Exp Res.

[41] Skelly MJ, Chappell AE, Carter E, Weiner JL (2015). Adolescent social isolation increases anxiety-like behavior and ethanol intake and impairs fear extinction in adulthood: Possible role of disrupted noradrenergic signaling. Neuropharmacology.

[42] Butler TR, Chappell AM, Weiner JL (2014). Effect of beta3 adrenoceptor activation in the basolateral amygdala on ethanol seeking behaviors. Psychopharmacology (Berl).

[43] Vashchinkina E (2012). GABA site agonist gaboxadol induces addiction-predicting persistent changes in ventral tegmental area dopamine neurons but is not rewarding in mice or baboons. J Neurosci.

[44] Walker QD, Rooney MB, Wightman RM, Kuhn CM (2000). Dopamine release and uptake are greater in female than male rat striatum as measured by fast cyclic voltammetry. Neuroscience.

[45] Blanchard BA, Steindorf S, Wang S, Glick SD (1993). Sex differences in ethanol-induced dopamine release in nucleus accumbens and in ethanol consumption in rats. Alcohol Clin Exp Res.

[46] Follesa P (2015). Chronic Intermittent Ethanol Regulates Hippocampal GABA(A) Receptor Delta Subunit Gene Expression. Front Cell Neurosci.

[47] Paxinos, G., & Franklin, K. B. J. The Mouse Brain in Stereotaxic Coordinates 2nd edn Academic Press: San Diego. CA, USA (2001).

[48] Schweizer C (2003). The gamma 2 subunit of GABA(A) receptors is required for maintenance of receptors at mature synapses. Mol Cell Neurosci.

